# A FRET-Based Ratiometric H_2_S Sensor for Sensitive Optical Molecular Imaging in Second Near-Infrared Window

**DOI:** 10.34133/research.0286

**Published:** 2023-12-28

**Authors:** Shan Lei, Kejia Jiang, Chenqing Zhang, Wei Sun, Yuantao Pan, Dong Wang, Peng Huang, Jing Lin

**Affiliations:** ^1^ Marshall Laboratory of Biomedical Engineering, International Cancer Center, Shenzhen Key Laboratory of Tumor Visualization Molecular Medicine, Laboratory of Evolutionary Theranostics (LET), School of Biomedical Engineering, Shenzhen University Medical School, Shenzhen University, Shenzhen 518055, China.; ^2^ Center for AIE Research, College of Materials Science and Engineering, Shenzhen University, Shenzhen 518060, China.

## Abstract

Second near-infrared (NIR-II) window optical molecular imaging kicks off a new revolution in high-quality imaging in vivo, but always suffers from the hurdles of inevitable tissue autofluorescence background and NIR-II probe development. Here, we prepare a Förster resonance energy transfer-based ratiometric NIR-II window hydrogen sulfide (H_2_S) sensor through the combination of an H_2_S-responsive NIR-II cyanine dye (acceptor, LET-1055) and an H_2_S-inert rhodamine hybrid polymethine dye (donor, Rh930). This sensor not only exhibits high sensitivity and selectivity, but also shows rapid reaction kinetics (~20 min) and relatively low limit of detection (~96 nM) toward H_2_S, allowing in vivo ratiometric NIR-II fluorescence imaging of orthotopic liver and colon tumors and visualization of the drug-induced hepatic H_2_S fluctuations. Our findings provide the potential for advancing the feasibility of NIR-II activity-based sensing for in vivo clinical diagnosis.

## Introduction

Activity-based sensing (ABS), an indispensable diagnostic technique, has a great potential to satisfy the strict criteria of biomedical research and clinical diagnosis when coupled with an appropriate imaging modality for visualization of living subjects [1–4]. Although numerous molecular imaging modalities, such as magnetic resonance imaging [5,6], photoacoustic imaging [7,8], and positron emission tomography [9], have afforded integrating ABS for sensitive imaging of disease-related biomarkers, the high cost and complex operation limit their applications in monitoring the feedback of biomarkers in vivo. In this context, fluorescence (FL) imaging technology holds a great utility and promise for in vivo diagnosis [10]. In particular, the FL imaging in the second near-infrared (NIR-II, 900 to 1,700 nm) window has revolutionized in vivo bioinformatics visualization [11–13], owing to its high sensitivity and spatiotemporal resolution [14–16], being employed in NIR-II FL imaging-guided surgery [17,18], in vivo tracking [19,20], and molecular event monitoring [21,22]. However, the excitation light-induced tissue autofluorescence background and light scattering always inevitably compromised the imaging definition and specificity in living systems. On account of this, in order to improve the imaging quality and signal-to-background ratio (SBR), the chemiluminescence resonance energy transfer (CRET) [23], bioluminescence resonance energy transfer (BRET) [24], or Förster resonance energy transfer (FRET) [25] effect has been introduced to construct NIR-II ratiometric imaging platforms. Among these energy transfer effects, the FRET, a potential self-calibration approach, is conducted by one fluorophore (donor) transferring its excited-state energy to another fluorophore (acceptor), affording the feasibility for precisely quantitative detection [26]. Benefiting from the tailored and precise molecular structures of donors or acceptors, FRET can endow NIR-II ratiometric imaging platforms with high sensitivity and specificity for various disease-related biomarker visualization.

Hydrogen sulfide (H_2_S), a mammalian gaseous messenger molecule, has been defined as a gasotransmitter due to its prominent biological role, akin to nitric oxide and carbon monoxide [27]. The physiological generation of H_2_S is mainly catalyzed by 2 pyridoxal-5′-phosphate-dependent enzymes (cystathionine β-synthase [CBS] and cystathionine γ-lyase [CSE]) responsible for metabolizing L-cysteine (L-Cys) [28]. Although originally considered toxic, H_2_S has been implicated in mediating various biological processes, including the modulation of inflammatory response [29], blood pressure [30,31] and metabolism [32], suppression of immune microenvironment [33], and treatment of various diseases (organ injury, cancers) [34–36]. In line with these findings, the accurate measurement of H_2_S level in living systems can reflect the state of physiology and pathology, thus deepening the knowledge of H_2_S-related biological information of diseases. Of note, H_2_S is highly reactive in living subjects due to its high nucleophilicity and reducibility [37], enabling the design of specific H_2_S probes via organic fluorophore platforms, akin to other biomarkers such as glutathione (GSH) [38,39] and reactive oxygen species [40–42]. Therefore, extensive efforts have been devoted to exploring the ABS for the diagnosis of various H_2_S-related diseases. To date, numerous H_2_S-activated FL probes have been developed based on boron-dipyrromethene [43,44], electrochromic materials [45,46], xanthene derivatives [47–49], and cyanine dyes [48,50] via the mechanism of nucleophilic addition, reduction, or thiolysis. However, most of these fluorophores exhibit emission wavelength in the first NIR (NIR-I) region or need complex and difficult probe chemistry. This is an urgent need for the accurate detection of the H_2_S level in various diseases in the NIR-II window.

**Fig. 1.**
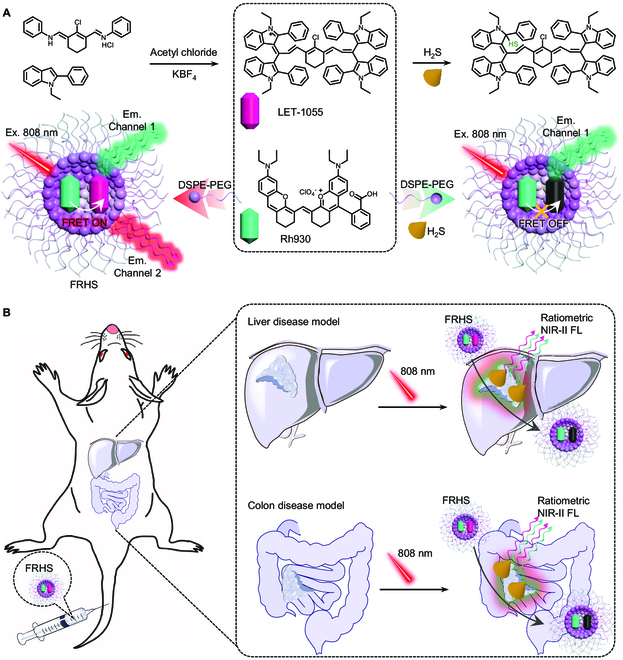
FRHS in NIR-II window biosensing. (A) Schematic illustration of the construction of the FRET-based ratiometric NIR-II window H_2_S sensor and (B) its application in sensitive visualization of H_2_S in vivo.

In this work, we constructed a FRET-based ratiometric NIR-II window H_2_S sensor (FRHS) through the combination of 2 NIR-II fluorophores (Fig. 1A). One is a NIR-II cyanine dye (LET-1055), a promising acceptor, which could be specifically responsive to H_2_S. The other is a rhodamine hybrid polymethine dye (Rh930) [51], a chemically stable donor, which not only exhibits sufficient overlap in emission/absorption bands between the 2 emitters, but also shows excellent inertia toward H_2_S. Therefore, the LET-1055 is capable of controlling the ratiometric NIR-II FL imaging by integrating with Rh930 based on the FRET mechanism. As expected, the FRHS can be sensitively and selectively activated by H_2_S over other reactive species to produce ratiometric NIR-II FL signals, and show rapid reaction kinetics and relatively low limit of detection (LOD, 96 nM) toward H_2_S, thus allowing ratiometric NIR-II FL imaging of orthotopic liver and colon tumors, and efficient monitoring of the lipopolysaccharide (LPS)- or S-adenosyl-L-methionine (SAM)-induced hepatic H_2_S fluctuations (Fig. 1B). Together, these lines of results indicate that FRHS holds great feasibility for sensitive in vivo diagnosis of H_2_S-related diseases through the potential NIR-II ABS.

## Results

### Synthesis and characterization of second near-infrared window H_2_S probe

**Fig. 2.**
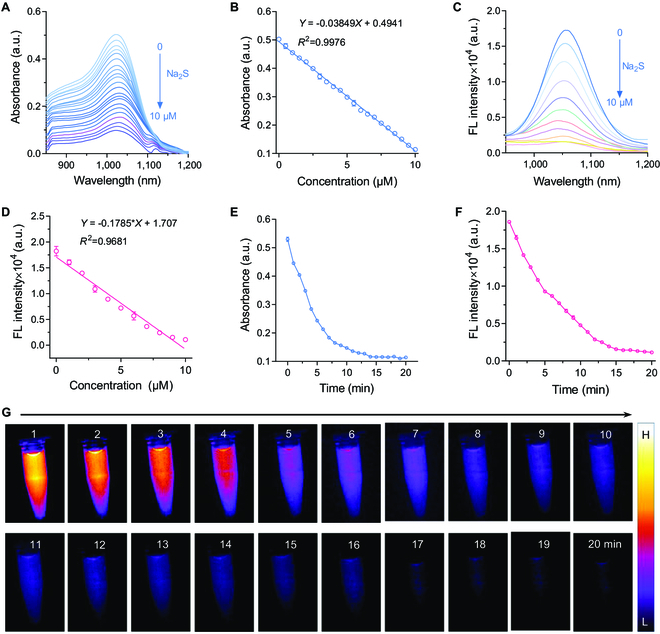
(A) UV–Vis–NIR spectra of LET-1055 (10 μM) after incubation with various concentrations of Na_2_S (0 to 10 μM) at 37 °C. (B) Corresponding NIR-II absorbance at 1,010 nm in (A). (C) NIR-II FL spectra of LET-1055 (10 μM) after incubation with various concentrations of Na_2_S (0 to 10 μM) at 37 °C. (D) Corresponding NIR-II emission at 1,055 nm in (A). Time-dependent NIR-II absorption (E) and FL (F) intensities of LET-1055 (10 μM) at 1,010 and 1,055 nm, respectively, after incubation with Na_2_S (10 μM) for 20 min. (G) Time-dependent NIR-II FL images (long pass filter: 1,100 nm) of LET-1055 (10 μM) upon incubation with Na_2_S (10 μM) for 20 min. Laser irradiation: 808 nm, 1 W cm^−2^. Data are presented as mean ± SD (*n* = 3).

To enable efficient response to H_2_S in the NIR-II window, a cyanine dye (LET-1055) with FL emission in the state-of-the-art NIR-II region was synthesized based on heptamethine structure. All the relevant compounds were characterized by a ^1^H nuclear magnetic resonance (NMR) spectrometer and electrospray ionization mass spectroscopy (Figs. S1 to S3). The maximum absorbance/emission of LET-1055 was 1,010/1,055 nm in dichloromethane with a relatively high quantum yield of 0.88% (Figs. S4 and S5). Of note, after reaction with H_2_S, the nucleophilic addition reaction between HS^−^ and the benzopyrrole group in LET-1055 could significantly quench its NIR-II emission, which can be recovered with the addition of Cu^2+^ (Fig. S6). Encouraged by this property, we next evaluated the sensing capability of LET-1055 to H_2_S. With the addition of different concentrations of Na_2_S in LET-1055 solution, the NIR-II absorption and emission bands showed obvious decrease tendency (Fig. 2A to D). Furthermore, the investigation of time-dependent reaction kinetics of LET-1055 with S^2−^ exhibited fast decrease of absorbance at 1,010 nm (Ab_1010_) and FL intensity at 1,055 nm (FL_1055_), which reached the minimum level within 15 min (Fig. 2E and F). This reaction process could also be visualized by NIR-II FL imaging system in a real-time manner (Fig. 2G). These results revealed that the LET-1055 possessed rapid reaction kinetics toward S^2−^. Subsequently, the selectivity of LET-1055 was performed. As a result, the LET-1055 was inert to other interferences (K^+^, Na^+^, Ca^2+^, Mn^2+^, Co^2+^, Fe^2+^, Cl^−^, HSO_3_^−^, SO_4_^2−^, S_2_O_3_^2−^, L-Arg, Vc, and GSH), but only could be specifically responsive to S^2−^ (Fig. S7). Taken together, these results indicated that LET-1055 was a specific H_2_S-responsive NIR-II fluorophore.

### Construction of a FRET-based ratiometric NIR-II window H_2_S sensor

**Fig. 3.**
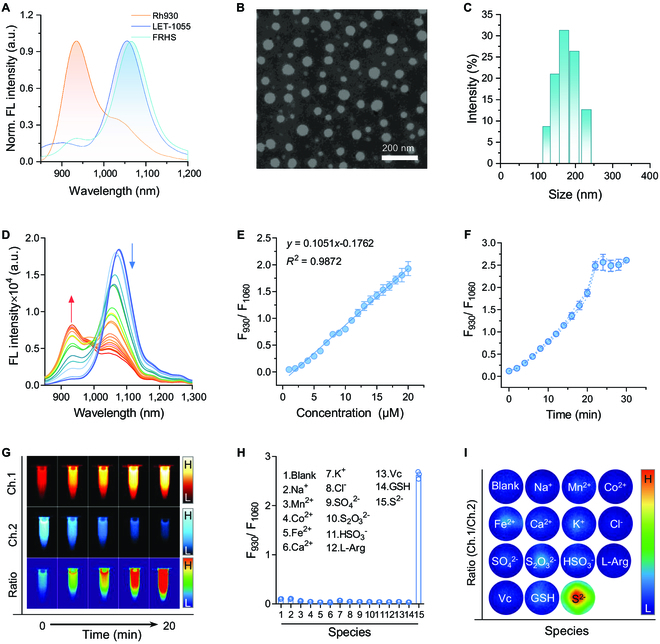
Synthesis and characterization of FRHS. (A) NIR-II FL spectra of Rh930, LET-1055, and FRHS. TEM image (B) and DLS analysis (C) of FRHS. (D) NIR-II FL spectra of FRHS after incubation with various concentrations of Na_2_S (0 to 20 μM) in PBS (10 mM, pH 6.8) at 37 °C. (E) Corresponding NIR-II FL emission (FL_930_/FL_1060_) ratio in (D). (F) Time-dependent NIR-II FL emission (FL_930_/FL_1060_) ratio of FRHS (containing 5 μM of LET-1055) after incubation with Na_2_S (10 μM) at 37 °C for different times. (G) NIR-II FL images at Ch.1 and Ch.2, and ratiometric Ch.1/Ch.2 FL images of FRHS (containing 5 μM of LET-1055) upon incubation with the indicated concentration of Na_2_S for 20 min. (H) NIR-II FL emission (FL_930_/FL_1060_) ratio of FRHS (containing 5 μM of LET-1055) upon incubation with different spices (Na^+^, Mn^2+^, Co^2+^, Fe^2+^, Ca^2+^, K^+^, Cl^−^, SO_4_^2−^, S_2_O_3_^2−^, HSO_3_^−^, L-Arg, Vc, GSH, and S^2−^) for 20 min. (I) Corresponding ratiometric Ch.1/Ch.2 FL images in (H). Data are presented as mean ± SD (*n* = 3).

To improve the sensitivity of LET-1055 to H_2_S, we strategically designed a FRET platform, where LET-1055 with H_2_S-quenched property acted as an energy acceptor, and the Rh930 with maximum absorbance/emission of 890/930 nm displayed as an energy donor (Fig. 3A), because of its sufficient overlap in absorbance/emission bands between these 2 emitters and excellent inertia toward H_2_S (Fig. S8). Therefore, Rh930 can not only be a credible energy donor to LET-1055, but also resist the targeted signal interference, ensuring the accuracy of H_2_S detection. Afterward, we constructed FRHS via amphiphilic 1,2-distearoyl-sn-glycero-3-phosphoethanolamine-*N*-[methoxy (polyethylene glycol)-2000] (DSPE-PEG_2000_)-assisted encapsulation of Rh930 and LET-1055 with a molar ratio of 1:2.5 (Rh930:LET-1055) [52]. Obviously, the as-prepared FRHS displayed the characteristic absorption/emission bands of Rh930 and LET-1055 (Fig. 3A and Fig. S9). The transmission electron microscopy (TEM) image showed that FRHS was dominated by spherical morphology with an average size of about 100 nm (Fig. 3B). The size distribution of FRHS was also evaluated by dynamic light scattering (DLS) (Fig. 3C). As a result, the hydrodynamic diameter of FRHS was about 170 nm, which is larger than that of TEM measurement. The size difference may be attributed to the DSPE-PEG_2000_ coating layer and the shrinking of the FRHS during the dryness process of TEM sample preparation. Subsequently, we evaluated the sensitivity and specificity of FRHS toward H_2_S. As shown in Fig. 3D, with the addition of S^2−^, the NIR-II FL intensity of FRHS was enhanced at 930 nm (FL_930_) and decreased at 1,060 nm (FL_1060_), yielding a good linear correlation between FL_930_/FL_1060_ intensities and S^2−^ concentrations from 0 to 20 μM (Fig. 3E). The LOD for S^2−^ was calculated to be 96 nM, which was lower than that in healthy tissues [45], thus enabling us to monitor H_2_S level in pathological tissues. Furthermore, the time-dependent reaction kinetics of FRHS with S^2−^ indicated that the FRHS also held fast reaction kinetics toward S^2−^ (Fig. 3F and Fig. S10), akin to the sole LET-1055. Encouraged by the above results, we applied NIR-II FL imaging to visualize the aforementioned FRET process. Obviously, the NIR-II FL intensity of channel 2 (Ch.2, 1,000 to 1,700 nm, filter: long-pass [LP] 1,100 nm) exhibited continuous decrease behavior for FRHS during incubation with S^2−^ solution, while the signals in channel 1 (Ch.1, 900 to 1,000 nm, filters: short-pass [SP] 1,000 nm and LP 900 nm) showed an opposite tendency. Thus, the ratiometric NIR-II FL imaging was delineated by the ratio of 2 channels (denoted as Ch.1/Ch.2) (Fig. 3G), showing a distinct FL signal enhancement. Combined with the excellent selectivity of FRHS for S^2−^ (Fig. 3H and I and Fig. S11), the S^2−^-induced ratiometric NIR-II FL imaging by FRHS possessed great potential for H_2_S detection.

### Ratiometric NIR-II FL imaging of H_2_S in living cells

**Fig. 4.**
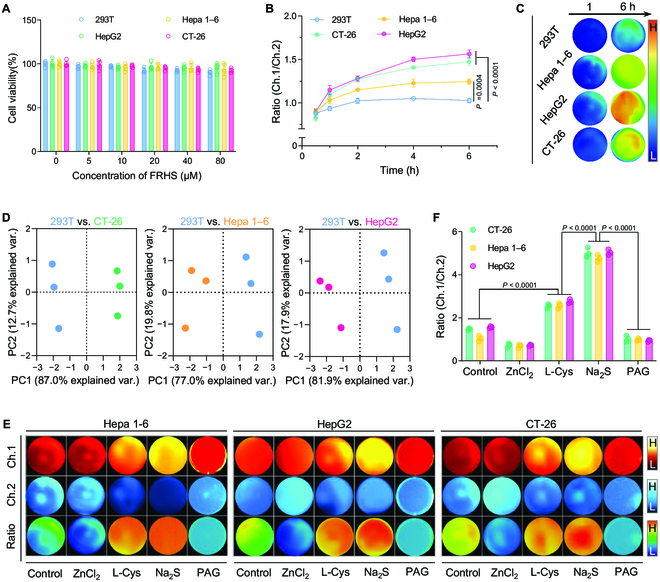
The feasibility of ratiometric NIR-II FL imaging of H_2_S in living cells. (A) Cell viability of 293T, Hepa 1-6, HepG2, and CT-26 cells after incubation with FRHS containing different concentrations of LET-1055 (0, 5, 10, 20, 40, and 80 μM) for 24 h. Data are presented as mean ± SD (*n* = 5). (B) Time-dependent ratiometric Ch.1/Ch.2 FL intensities of 293T, Hepa 1-6, HepG2, and CT-26 cells after incubation with FRHS for different times. (C) Corresponding ratiometric Ch.1/Ch.2 FL images in (B). (D) PCA of FRHS for 293T, Hepa 1-6, HepG2, and CT-26 cells. (E) Ratiometric Ch.1/Ch.2 FL images of FRHS in Hepa 1-6, HepG2, and CT-26 cells with different treatments (PBS, ZnCl_2_, L-Cys, Na_2_S, and PAG). (F) Quantification of Ch.1/Ch.2 signals from (E). Data are presented as mean ± SD (*n* = 3). Statistical significance was calculated via one-way ANOVA with Tukey’s multiple comparisons test.

Benefiting from high sensitivity and selectivity of FRHS toward H_2_S, we next evaluated the reliability of FRHS for ratiometric NIR-II FL imaging of H_2_S in vitro (Fig. S12A). The liver (HepG2 and Hepa 1-6) and colon cancer (CT-26) cell lines were chosen for H_2_S-level investigation, due to their overexpression of CBS and CSE for the biosynthesis of endogenous H_2_S [45,53,54]. Meanwhile, the normal cell line of HEK293T was chosen as a control group. The cell viability by methyl thiazolyl tetrazolium (MTT) assay indicated that FRHS had a superior biocompatibility for these cell lines even at the high concentration of 80 μM (quantified by LET-1055) (Fig. 4A). Next, we acquired the NIR-II FL signals in 2 channels to map the ratiometric FL signals. After incubation with FRHS for 6 h, the FL signals in HepG2, Hepa 1-6, and CT-26 cells showed 1.6-, 1.2-, and 1.5-fold enhancement compared to that in normal cells (HEK293T), respectively (Fig. 4B and C and Fig. S12B to E). Meanwhile, principal components analysis (PCA) revealed that the FRHS distinctly separated normal cells and cancer cells with relatively high intracellular H_2_S level (Fig. 4D). To further demonstrate the reliability of FRHS for H_2_S detection in vitro, we subsequently investigated the H_2_S fluctuation in cancer cells. As shown in Fig. 4E, the ratiometric FL signals of cancer cells were substantially decreased when pre-treated with ZnCl_2_ (a scavenger of H_2_S). In contrast, after the pre-treatment of Hepa 1-6 cells with extraneous Na_2_S and the treatment of FRHS, the ratiometric Ch.1/Ch.2 FL signals showed about 4.2-fold enhancement compared to that of control group. Furthermore, the L-Cys, a precursor for the biosynthesis of H_2_S, was utilized to upregulate the intracellular H_2_S levels [36], followed by the incubation of FRHS. As a result, the obvious Ch.1/Ch.2 FL signals up to 2.5-fold of the control group were observed, which could be efficiently suppressed by the addition of DL-propargylglycine (PAG; 50 μg ml^−1^) to inhibit the activity of endogenous CSE [29,45]. Meanwhile, the similar fluctuation tendencies of H_2_S were observed in HepG2 and CT-26 cells (Fig. 4E). Taken together, these results strongly demonstrated that the FRHS is capable of highly specific and sensitive detecting endogenous H_2_S in cancer cells.

### Ratiometric NIR-II FL imaging of H_2_S in orthotopic liver tumors

**Fig. 5.**
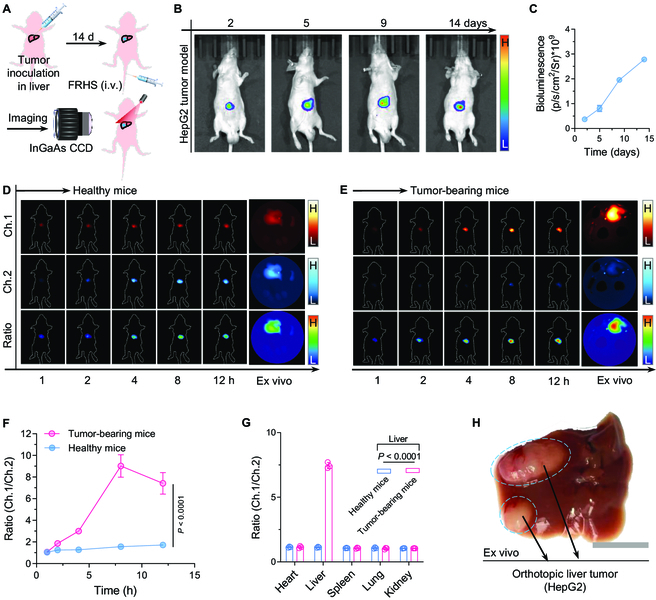
In vivo ratiometric NIR-II FL imaging of H_2_S in orthotopic liver tumor. (A) Schematic illustration for establishment of the orthotopic liver tumor-bearing mouse model and imaging experiment. (B) Bioluminescence images of HepG2/Luc inoculated mice at different time points (2, 5, 9, and 14 days). (C) Quantification of bioluminescence signals from (B). Data are presented as mean ± SD (*n* = 3). (D and E) NIR-II FL images at Ch.1 and Ch.2, and ratiometric Ch.1/Ch.2 FL images of healthy mice (D) and tumor-bearing mice (E), and corresponding ex vivo organs. (F and G) Quantification of ratiometric Ch.1/Ch.2 signals of mice after various treatments of in vivo (F) and ex vivo organs (G). Data are presented as mean ± SD (*n* = 3). Statistical significance was calculated via two-tailed Student’s *t* test. (H) Optical image of ex vivo liver (blue dashed circles: orthotopic liver tumor nodules). Scale bar is 1 cm. *n* = 3 independent experiments.

Encouraged by the good performance of FRHS for H_2_S detection in vitro, we evaluated the feasibility of FRHS for ratiometric NIR-II FL imaging of H_2_S in orthotopic liver tumors. As shown in Fig. 5A, the luciferase-transfected HepG2 cells (HepG2/Luc) were inoculated into the lube of livers of mice to establish orthotopic liver tumors. After 14 days, the strong bioluminescence (BL) signals in livers were collected by the IVIS Spectrum imaging system, indicating the successful construction of orthotopic liver tumors (Fig. 5B and C). Subsequently, the FRHS was intravenously injected into mice, followed by the 808-nm laser irradiation (0.3 W cm^−2^) at different time points (1, 2, 4, 8, and 12 h). As a comparison, the healthy mice were subjected to similar treatments. The NIR-II FL signals were collected in Ch.1 and Ch.2, respectively (Fig. 5D and E). Obviously, for the healthy mice group, the signals in Ch.1 and Ch.2 showed a similar increasing trend, resulting in the inconspicuous ratiometric signal variation in mice body and ex vivo major organs (heart, liver, spleen, lung, and kidney) (Fig. 5D). However, for the tumor-bearing mice group, the NIR-II signals showed distinct enhancement in Ch.1 at 8 h post-injection (p.i.), and exhibited a very weak increase in Ch.2, receiving a distinct ratiometric signal variation, thus indicating that the endogenous H_2_S generated by liver tumor cells could be effectively detected by FRHS (Fig. 5E). Of note, the ratiometric NIR-II FL signals of in vivo and ex vivo livers showed 5.8-fold (Fig. 5F) and 6.3-fold (Fig. 5G) enhancements, respectively, compared to that of healthy mice. Combined with the optical image of ex vivo liver tissue (Fig. 5H, blue dashed circles: orthotopic liver tumor nodules), the above results demonstrated that FRHS had the feasibility of in vivo ratiometric NIR-II FL imaging of H_2_S. In addition, no pathological abnormality or inflammation was observed in slices of major organs (heart, liver, spleen, lung, and kidney) from FRHS-treated healthy mice (Fig. S13A). Meanwhile, no abnormal index was detected in blood biochemistry analysis (Fig. S13B to E). These results demonstrated that FRHS possessed good biocompatibility.

To further confirm the reliability of FRHS for ratiometric NIR-II FL imaging of H_2_S, we constructed murine orthotopic liver tumors by using luciferase-transfected Hepa 1-6 cells (Hepa 1-6/Luc) (Fig. S14A), which was confirmed by the strong BL signals in liver after 14 days of inoculation (Fig. S14B and C). After intravenous injection of FRHS, followed by the 808-nm laser irradiation (0.3 W cm^−2^) at different time points (1, 2, 4, 8, and 12 h), the NIR-II FL signals in Ch.1 and Ch.2 showed a time-dependent increasing trend in healthy mice, which could be attributed to the metabolism of FRHS in the livers (Fig. S14D). On the contrary, the ratiometric Ch.1/Ch.2 NIR-II FL signals of tumor-bearing mice were 4.8-fold (in vivo livers, Fig. S14E and F) and 6.5-fold (ex vivo livers, Fig. S14G) higher, respectively, than those of healthy mice. The observation is consistent with the results obtained from orthotopic HepG2 tumor-bearing mice. Combined with the optical image of ex vivo liver tissue (Fig. S14H, blue dashed circles: orthotopic liver tumor nodules), the above results indicated that FRHS is capable of effective detection of H_2_S in liver tumors via ratiometric NIR-II FL imaging.

### Ratiometric NIR-II FL imaging of H_2_S in orthotopic colon tumor

**Fig. 6.**
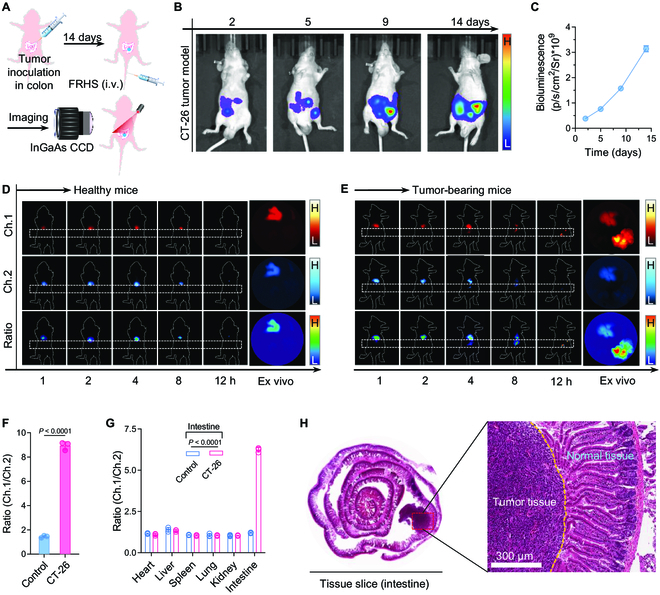
In vivo ratiometric NIR-II FL imaging of H_2_S in orthotopic colon tumor. (A) Schematic illustration for establishment of the orthotopic colon tumor-bearing mouse model and imaging experiment. (B) Bioluminescence images of CT-26/Luc inoculated mice at different time points (2, 5, 9, and 14 days). (C) Quantification of bioluminescence signals from (B). Data are presented as mean ± SD (*n* = 3). (D and E) NIR-II FL images at Ch.1 and Ch.2, and ratiometric Ch.1/Ch.2 FL images of healthy mice (D) and tumor-bearing mice (E), and corresponding ex vivo organs. The white dashed box: colons. (F and G) Quantification of ratiometric Ch.1/Ch.2 signals of mice after various treatments of in vivo (F) and ex vivo organs (G). Data are presented as mean ± SD (*n* = 3). Statistical significance was calculated via 2-tailed Student’s *t* test. (H) H&E staining of ex vivo intestine. Red dashed box: colon tumor nodule. *n* = 3 independent experiments.

In addition to liver cancers, colorectal cancers are also overexpressing CBS and CSE for the biosynthesis of endogenous H_2_S [53,54], which can regulate angiogenesis and cell proliferation. Therefore, the visualization of H_2_S in colorectal cancers is of great importance to reveal the related pathological information. Afterward, orthotopic CT-26 colon tumors were established by luciferase-transfected CT-26 cells (CT-26/Luc) (Fig. 6A to C). The ratiometric NIR-II FL imaging of H_2_S in colon tumors was recorded on a NIR-II FL imaging system. Along the same lines, the healthy mice were chosen as the control group, followed by intravenous injection of FRHS. Obviously, for healthy mice, under the 808-nm laser irradiation (0.3 W cm^−2^), the NIR-II FL signals cannot be found in colon (Fig. 6D, white dotted boxes), and were only observed in livers, mainly attributed to the normal metabolism of FRHS. Fortunately, for CT-26 tumor-bearing mice, the NIR-II signals showed distinct enhancement in Ch.1 at 12 h p.i. of FRHS, and exhibited a weak increase in Ch.2, receiving a distinct ratiometric Ch.1/Ch.2 FL signal variation in colon (Fig. 6E). Of note, the ratiometric NIR-II FL signals were 6.0-fold (in vivo colons, Fig. 6F) and 5.2-fold (ex vivo colons, Fig. 6G), higher, respectively, than those of healthy mice. The hematoxylin–eosin (H&E) staining images of intestine showed a distinct area of CT-26 tumor (Fig. 6H). On the basis of the above analysis, the FRHS is capable of ratiometric NIR-II FL imaging of H_2_S in colon tumors, thus deepening our understanding and interrogation of H_2_S-related biological information of colon tumors.

### Ratiometric NIR-II FL imaging of H_2_S in drug-induced liver injury

**Fig. 7.**
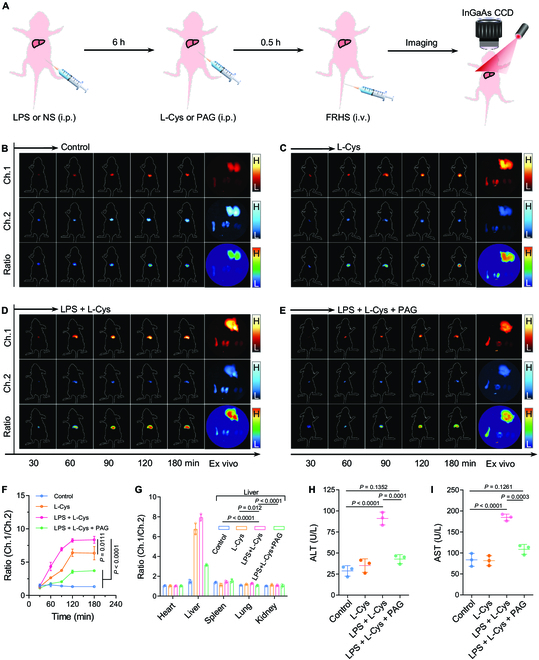
In vivo ratiometric NIR-II FL imaging of H_2_S in LPS-induced liver injury. (A) Schematic illustration for the establishment of the liver-injured mouse model and imaging experiment. (B to E) NIR-II FL images at Ch.1 and Ch.2, and ratiometric Ch.1/Ch.2 FL images of mice with PBS (B), L-Cys (C), LPS + L-Cys (D), or LPS + L-Cys + PAG (E) treatment, and the corresponding ex vivo organs. (F and G) Quantification of ratiometric Ch.1/Ch.2 signals of mice after various treatments of in vivo (F) and ex vivo organs (G). ALT (H) and AST (I) levels in the blood serum of mice after different treatments. Data are presented as mean ± SD (*n* = 3). Statistical significance was calculated via one-way ANOVA with Tukey’s multiple comparisons test.

Apart from the cancers, the H_2_S is also an active signaling indicator, which closely associates with various liver diseases. Therefore, the detection of hepatic H_2_S level is of great significance for early diagnosis and understanding of liver diseases [46]. Among these diseases, the drug-induced liver injury has attracted much attention. In particular, the LPS-induced liver inflammation exhibited an up-regulated expression of CSE [54]. On account of this, we established an LPS-induced liver inflammation model (Fig. 7A to G). Firstly, the mice were randomly divided into 4 groups (Control, L-Cys, LPS + L-Cys, and LPS + L-Cys + PAG). For the control group, the mice were intraperitoneally injected with saline (100 μl), followed by the intravenous injection of FRHS (5 mg kg^−1^) at 6.5 h p.i. After that, the mice were imaged by the NIR-II FL imaging system (Fig. 7A). As shown in Fig. 7B, the FL signals in Ch.1 and Ch.2 gradually increased over time, indicating the efficient accumulation of FRHS in the livers of mice. To further validate the activation of ratiometric FL signals by H_2_S in livers, mice were intraperitoneally injected with L-Cys (100 μl, 1 mM) to upregulate the production of hepatic H_2_S, followed by intravenous injection of FRHS (5 mg kg^−1^). Of note, the NIR-II FL signals showed a distinct time-dependent enhancement in Ch.1, but exhibited weak variation in Ch.2. Therefore, the obviously ratiometric Ch.1/Ch.2 NIR-II FL signals were observed in livers of mice and showed 4.9-fold enhancement compared to that of healthy mice at 3 h p.i. (Fig. 6C). In addition, among the mice pre-injected with LPS (2 mg kg^−1^), followed by the intraperitoneal injection of L-Cys (100 μl, 1 mM) at 6.5 h p.i. of FRHS, the strongest ratiometric Ch.1/Ch.2 NIR-II FL signals were observed in vivo and ex vivo (Fig. 7D). The signals were 6.3-fold (in vivo livers, Fig. 7F) and 6.0-fold (ex vivo livers, Fig. 7G) higher, respectively, than those of healthy mice. Moreover, when the mice were intraperitoneally injected with PAG (5 mg kg^−1^) to suppress the production of H_2_S through inhibition of CSE activity, the ratiometric Ch.1/Ch.2 NIR-II FL signals were significantly decreased in livers both in vivo and ex vivo (Fig. 6E to G). In order to confirm the degree of liver damage, we evaluated the activities of 2 important hepatic indicators, aspartate aminotransferase (AST) and alanine aminotransferase (ALT). As shown in Fig. 7H and I, the AST and ALT levels in LPS-treated mice were significantly elevated and can be efficiently suppressed after pre-treatment with PAG. These lines of evidence indicated that FRHS is useful for the sensitive detection of hepatic H_2_S level in liver-injured mice.

To further demonstrate the feasibility of FRHS for hepatic H_2_S-sensitive detection, we applied SAM, a CBS activator, to elevate the endogenous H_2_S level in liver [50]. The mice were intravenously injected with FRHS (5 mg kg^−1^) at 12 h p.i. of SAM (Fig. S15A). The FL images revealed that the ratiometric NIR-II FL signals in the liver of SAM-injected mice were much higher than that of the saline group, implying that SAM injection could enhance the H_2_S level (Fig. S15B to D). Of note, the FL images of isolated viscera disclosed the liver accumulation of FRHS, and the ratiometric Ch.1/Ch.2 NIR-II FL signals in the livers of SAM-injected mice exhibited 8.8-fold enhancement compared to that of non-SAM-treated mice (Fig. S15E), indicating that SAM injection induced endogenous H_2_S enhancement in the livers. On the basis of the above analysis, the FRHS was capable of sensitive ratiometric NIR-II FL imaging of H_2_S in living mice.

## Discussion

To date, cyanine or hemicyanine fluorophore-based ABSs have attracted much attention in disease diagnosis by PA or FL imaging technology [41,50,55], due to the easily tailored and precise molecular structure of these fluorophores and relatively high imaging sensitivity of ABS. In order to further improve the imaging quality and SBR, various imaging strategies including afterglow imaging [42,56,57] and the introduction of CRET [23], BRET [24], or FRET [25] effect were integrated into ABS platforms. In addition, the light localized in the NIR-II window possessed superior deep tissue penetration ability, which was strongly evidenced by transcranial neuromodulation [58,59], showing great potential for various disease-related biomarker visualization in vivo. Therefore, the combination of NIR-II PA or FL imaging technology and ABS platform is a promising pathway for sensitive in vivo diagnosis of H_2_S-related diseases.

In this study, we constructed a FRET-based ratiometric NIR-II window H_2_S sensor (FRHS) through the combination of LET-1055 and Rh930 for sensitive and precise in situ imaging of H_2_S in various diseases. Of note, the LET-1055 exhibited intense NIR-II emission, which could be selectively quenched by H_2_S but inert to other reactive species. In addition, the LET-1055 was capable of controlling the ratiometric NIR-II FL imaging by introducing Rh930 with excellent inertia toward various reactive species. Significantly, the FRHS could be sensitively and selectively activated by H_2_S to generate ratiometric NIR-II FL images, and showed rapid reaction kinetics toward H_2_S under physiological conditions. Meanwhile, FRHS possessed a relatively low LOD (96 nM), allowing ratiometric NIR-II FL imaging of orthotopic liver and colon tumors, and visualization of the H_2_S level in LPS-induced liver injury and SAM-induced hepatic H_2_S fluctuations, thus deepening our understanding of H_2_S-related diseases in vivo. Therefore, our work highlights the potential of FRHS for in vivo H_2_S detection/imaging.

## Materials and Methods

### Materials and characterization

Potassium tetrafluoroborate, 1-ethyl-2-phenylindole, acetyl chloride, acetic anhydride, dimethyl sulfoxide (DMSO), dichloromethane, and methanol were purchased from Energy Chemical unless otherwise stated. 3-(4,5-Dimethylthiazol-2-yl)-2,5-diphenyltetrazolium bromide (MTT), LPS, SAM, and penicillin–streptomycin solution (100×) were purchased from Beyotime Institute of Biotechnology (Shanghai, China). Fetal bovine serum (FBS) and Dulbecco’s modified Eagle’s medium (DMEM) were purchased from GIBCO Co., Ltd. All cell lines were purchased from Cell Bank of the Chinese Academy of Sciences (Shanghai, China). The morphologies of the prepared FRHS nanoprobes were observed by TEM (Hitachi H-7700, 100 KV, Japan). DLS analysis was performed on Zetasizer Nano-ZS90 (Malven, UK). NMR spectra were measured on a Bruker Avance 400 MHz NMR spectrometer (Bruker, Switzerland). Electrospray ionization mass spectra were recorded on a mass spectrometer (Q-Exactive, USA). Ultraviolet–visible–near-infrared (UV–Vis–NIR) spectra were obtained on a Lambda 1050+ spectrometer (PerkinElmer, America). NIR-II FL spectra were obtained using an FLS1000 (Edinburgh Instruments, UK). NIR-II FL imaging was performed on Princeton Instrument NIR640 (USA).

### Synthesis of Rh930 and LET-1055

The Rh930 was synthesized according to a previous report [51]. For LET-1055, the 1-ethyl-2-phenylindole (4.3 mmol, 1.14 g) and acetyl chloride (4.0 mmol, 884 mg) in acetic anhydride (20 ml) was heated at 55 °C for 4 h. N-[(3-(Anilinomethylene)-2-chloro-1-cyclohexen-1-yl)methylene] aniline monohydrochloride (1.0 mmol, 359 mg) and potassium tetrafluoroborate (1.1 mmol, 126 mg) were added to the mixed solution and then heated at 100 °C for 1.5 h. After cooling, the solvent was removed, and the product was purified with silica gel column chromatography with dichloromethane/methanol (20:1, v/v) as the eluent to obtain the black solid (yield 33%). ^1^H NMR (400 MHz, DMSO-d_6_) δ 8.14 (s, 2H), 7.78 to 7.67 (m, H), 7.63 to 7.52 (m, 6H), 7.33 to 7.17 (m, 22H), 7.07 to 7.03 (t, 3H), 6.75 to 6.71 (m, 1H), 6.48 to 6.46 (d, 3H), 4.15 to 4.01 (m, 8H), 3.17 (s, 2H), 2.02 to 1.96 (m, 4H), 1.23 to 1.16 (m, 12H). MALDI-MS (ESI): calculated for 1,093.49, found for [M+ Na]^+^ = 1,093.54016.

### Sensitivity of LET-1055 toward H_2_S

In order to explore the relationship between the FL intensity of LET-1055 and the H_2_S concentration, 0.5 μM of Na_2_S solution (an H_2_S donor reagent commonly used in in vitro tests) was added dropwise to the LET-1055 solution (PBS, 10 mM, pH 7.4, containing 20% of DMSO) (10 μM). After each dropwise addition, followed by reacting at 37 °C for 2 min, the absorption and FL intensities of LET-1055 were measured, and linearly fitted with the various concentration of Na_2_S (0 to 10 μM). For the reaction kinetics exploration, 10 μM of Na_2_S was added into the LET-1055 solution, and the absorption and FL intensities of LET-1055 at 1,010 and 1,055 nm were measured every 1 min within 20 min, respectively. In addition, the corresponding NIR-II FL images were collected on a NIR-II FL imaging system under 808-nm laser irradiation (power density: 0.3 W cm^−2^, exposure time: 50 ms). Finally, the FL images and ratiometric FL images were processed by ImageJ software.

### Selectivity of LET-1055 toward H_2_S

In order to study the selectivity of LET-1055 to the H_2_S substrate and various interfering substances, 16 groups of LET-1055 solutions (PBS, 10 mM, pH 7.4, containing 20% of DMSO) (10 μM) were taken, and 3 samples were paralleled in each group. The first group of LET-1055 solution was not treated as a blank control, and the remaining 15 groups of LET-1055 solutions were mixed with Na^+^ (500 μM), Mn^2+^ (500 μM), Co^2+^ (500 μM), Fe^2+^ (500 μM), Ca^2+^ (500 μM), K^+^ (500 μM), Cl^−^ (500 μM), SO_4_^2−^ (500 μM), S_2_O_3_^2−^ (500 μM), HSO_3_^−^ (500 μM), L-Arginine (L-Arg, 500 μM), Vitamin C (Vc, 500 μM), glutathione (GSH, 500 μM), and S^2−^ (10 μM) followed by incubation at 37 °C for 20 min, and then the absorption spectra, FL spectra, and NIR-II FL imaging of all solutions were measured under 808-nm laser irradiation (power density: 0.3 W cm^−2^, exposure time: 50 ms). Finally, the FL images and ratiometric FL images were processed by ImageJ software.

### Chemical inertia of Rh930 toward H_2_S

The stability of Rh930 toward H_2_S was explored. After the addition of 10 μM of Na_2_S into the Rh930 solution, the absorption and FL intensity of Rh930 at 890 nm at 930 nm were measured every 2 min within 60 min under 808-nm laser irradiation (power density: 0.3 W cm^−2^, exposure time: 50 ms), respectively.

### Preparation of FRHS

All the nanoprobes were prepared using the amphiphilic polymer-assisted nanoprecipitation method [46]. Typically, Rh930 and LET-1055 (with a ratio of 1:2.5, w/w) were added into a 1-ml dichloromethane solution of DSPE-PEG_2000_ (10 mg). Then, the mixture was added dropwise into deionized water (5 ml), and the ultrasonic bath was continued for 20 min. The dichloromethane was removed by nitrogen sparging. The remaining aqueous solution was filtered through a syringe-driven filter (0.22 μm) (Millipore) and centrifuged at 3,500 rpm for 15 min at 4 °C using a 30-kDa ultrafiltration tube, and washed 3 times with deionized water. The FRHS was obtained and determined by FL spectroscopy. The concentration of LET-1055 contained in the obtained FRHS was determined according to its absorption at 1,010 nm, and the mass concentration is determined through sampling and freeze-drying. The final samples were stored at 4 °C in the dark.

### Sensitivity of FRHS toward H_2_S

Firstly, 0.5 μM of Na_2_S solution was added dropwise to the FRHS solution (containing 5 μM of LET-1055). After each dropwise addition, followed by reacting at 37 °C for 2 min, the absorption and FL spectra of FRHS were measured, respectively, and the FL ratio of FL_930_/FL_1060_ linearly fitted with the various concentration of Na_2_S (0 to 20 μM). For the reaction kinetics exploration, 10 μM of Na_2_S was added into the FRHS (containing 5 μM of LET-1055) solution, and the FL ratio of FL_930_/FL_1060_ was measured every 1 min within 20 min. In addition, the corresponding NIR-II FL images were collected on the NIR-II FL imaging system under 808-nm laser irradiation (power density: 0.3 W cm^−2^, exposure time: 50 ms). Finally, the FL images and ratiometric FL images were processed by ImageJ software.

### Selectivity of FRHS toward H_2_S

For the exploration of selectivity of FRHS (containing 5 μM of LET-1055) to the H_2_S substrate and various interfering substances, 16 groups of FRHS solutions (containing 5 μM of LET-1055) were taken, and 3 samples were paralleled in each group. The first group of FRHS solution was not treated as a blank control, and the remaining 15 groups of FRHS solutions were mixed with various reactive species, followed by incubation at 37 °C for 30 min, and then the variation of absorption and FL spectra for FRHS solutions were recorded. In addition, the ratiometric NIR-II FL imaging was conducted using the NIR-II FL imaging system under 808-nm laser irradiation (power density: 0.3 W cm^−2^, exposure time: 50 ms). Finally, the FL images and ratiometric FL images were processed by ImageJ software.

### Cell culture and animals

Human liver cancer HepG2 cells, human renal epithelial 293T cells, murine liver cancer Hepa 1-6 cells, and murine colon cancer CT-26 cells were purchased from Cell Bank of the Chinese Academy of Sciences (Shanghai, China). All cell lines were routinely tested to exclude infection with mycoplasma, and authenticated by the supplier using short tandem repeat test. All of the cells were cultured in DMEM. All the media were supplemented with 10% FBS and 1% penicillin and streptomycin. All cells were cultured at 37 °C with 5% CO_2._

Female and male BALB/C nude mice and C57BL/6 mice aged 5 to 6 weeks were purchased from Guangdong Medicinal Laboratory Animal Center (Guangzhou, China) and all animal experiments were carried out in strict accordance with the regulations of the Animal Ethical and Welfare Committee of Shenzhen University (AEWC-SZU, maximal tumor size: <1,000 mm^3^). All the experimental mice were housed under standard conditions (temperature: ~22 °C, humidity: 40% to 70%, 12-h dark–light cycles) with free access to sterile food and water.

### In vitro and in vivo biosafety evaluation of FRHS

The cytotoxicity of FRHS was evaluated on a series of cell lines (293T, HepG2, Hepa 1-6, and CT-26 cells). First, the cells were cultured in 96-well plates at 37 °C with 5% CO_2_ for 24 h. Then, the medium was removed, and the cells were exposed to various concentrations of FRHS (0, 5, 10, 20, 40, and 80 μM, quantified by LET-1055) for a further 24 h. Subsequently, cell viabilities were evaluated using the MTT assay. For in vivo biosafety evaluation, the mice were intravenously injected with PBS and FRHS solution, respectively. Then, the major organs (heart, liver, spleen, lung, and kidney) of mice were collected at 24 h p.i. and evaluated by histological analysis.

### In vitro ratiometric NIR-II FL imaging

For intracellular H_2_S imaging, 293T cells, Hepa 1-6 cells, HepG2 cells, and CT26 cells were seeded in 12-well plates (1×10^5^ cells/well) and cultured in DMEM for 24 h. Then, the medium was replaced with fresh medium containing FRHS (in which the concentration of LET-1055 is 20 μM), and further incubated for 1, 2, 3, 4 and 6 h, respectively. The imaging data could be collected under the 2 channels (Ch.1: SP 1,000 nm and LP 900 nm; Ch.2: LP 1,100 nm) corresponding to different time points, and statistical analysis was carried out.

In addition, in order to better understand the association between the imaging effect of FRHS and the expression level of H_2_S, experiments were performed according to the following procedure. The cells were seeded in 12-well plates (1×10^5^ cells/well) and incubated for 24 h. Each of the above 4 types of cells was divided into 5 groups for experiments: (a) Cells were incubated with fresh medium containing FRHS for 4 h; (b) cells were pretreated with 0.3 mM ZnCl_2_ for 10 min to remove endogenous H_2_S, and then incubated with fresh medium containing FRHS for 4 h; (c) to increase endogenous H_2_S in the H_2_S production group, the cells were incubated with 0.2 mM L-Cys for 1 h in advance, and then incubated with fresh medium containing FRHS for 4 h; (d) cells were incubated with fresh medium containing FRHS for 4 h, and then incubated in DMEM medium containing 1 mM Na_2_S for 1 h at 37 °C; (e) the PAG was added into the medium to inhibit the activity of CSE, followed by the incubation with fresh medium containing FRHS for 4 h. Then, the medium was discarded and washed once with cold PBS. Finally, we obtained imaging data under 2 channels corresponding to different groups and performed statistical analysis. NIR-II FL images and ratiometric NIR-II FL images were processed by ImageJ software.

### Orthotopic liver tumor mouse model

For the establishment of the orthotopic liver tumor, a midline incision was made on the front abdomen of mice, then 100 μl of luciferase-transferred Hepa 1-6 (Hepa 1-6/Luc, 2×10^6^) or HepG2 cells (HepG2/Luc, 2×10^6^) in serum-free medium was carefully injected into the liver lobe, and the wound was sutured. Tumor growth was monitored by bioluminescent imaging, followed by imaging exploration after 2 weeks.

### Orthotopic colon tumor mouse model

To establish orthotopic colon tumor, the Luciferase-transfected CT-26 cells (CT-26/Luc, 5×10^5^) in 50 μl of PBS were injected intraperitoneally into the colon of male nude mice, and then tumor growth was monitored by bioluminescent imaging, followed by imaging exploration after 2 weeks.

### LPS-induced liver injury

Female nude mice (5 to 6 weeks) were used to establish animal models. The mice were intraperitoneally injected with LPS (2 mg kg^−1^) for 2 h. At predefined time points, the mice were further intraperitoneally administered with L-Cys. After 30 min, mice were intravenously injected with the FRHS (5 mg kg^−1^). After that, the mice were used for NIR-II FL imaging.

### SAM-induced hepatic H_2_S fluctuations

Female nude mice (5 to 6 weeks) were used to establish animal models. According to the method reported in the literature [50], mice were intraperitoneally injected with SAM (200 μl, 100 mg kg^−1^) or normal saline (200 μl) 12 h in advance. After that, FRHS (10 mg kg^−1^) was injected into the mice through the tail vein, followed by use for NIR-II FL imaging.

### In vivo ratiometric NIR-II FL imaging

After the mice were intravenously injected with FRHS with a preset concentration, followed by 808-nm laser irradiation (power density: 0.3 W cm^−2^, exposure time: 500 ms), the mice were subjected to NIR-II FL imaging at different time points, and the corresponding wavelength filters were selected to collect imaging data under 2 channels (Ch.1: SP 1,000 nm and LP 900 nm; Ch.2: LP 1,100 nm). Following in vivo imaging, mice were dissected and major internal organ imaging was performed ex vivo. NIR-II FL images and ratiometric NIR-II FL images were processed by ImageJ software.

### Statistical analysis

All data represent the mean ± SD. One-way ANOVA with Tukey’s multiple comparisons was used for multiple comparisons when more than 2 groups were compared, and 1-tailed or 2-tailed Student’s *t* test was used for 2-group comparisons. All statistical differences were calculated by using GraphPad Prism 9.5 (GraphPad Software, Inc., CA, USA). In all types of statistical analysis, values of *P* < 0.05 were considered significant.

## Data Availability

The authors declare that the data supporting the findings of this study are available within the article and its Supplementary Materials.

## Supplementary Materials

Supplementary 1 Experimental Section Figs. S1 to S15
