# Supplementary material for: A FRET-Based Ratiometric H2S Sensor for Sensitive Optical Molecular Imaging in Second Near-Infrared Window
Source: Research (Wash D C). 2023 Dec 28;6:0286. doi: 10.34133/research.0286 (PMC10755252; doi:10.34133/research.0286)
Supplement: Supplementary 1 — Experimental Section Figs. S1 to S15 [file research.0286.f1.docx]

**A FRET-based Ratiometric H_2_S Sensor for Sensitive Optical Molecular Imaging in Second Near-Infrared Window**

Shan Lei^1^†, Kejia Jiang^1^†, Chenqing Zhang^1^, Wei Sun^1^, Yuantao Pan^1^, Dong Wang^2^, Peng Huang^1^, and Jing Lin^1^*

^1^Marshall Laboratory of Biomedical Engineering, International Cancer Center, Laboratory of Evolutionary Theranostics (LET), School of Biomedical Engineering, Shenzhen University Medical School, Shenzhen University, Shenzhen 518055, China

^2^Center for AIE Research, College of Materials Science and Engineering, Shenzhen University, Shenzhen 518060, China

*Address correspondence to: [jingl@szu.edu.cn](mailto:jingl@szu.edu.cn)

†These authors contributed equally to this work

**Experimental Section**

**Materials:** Potassium tetrafluoroborate, 1-ethyl-2-phenylindole, acetyl chloride, acetic anhydride, dichloromethane (DCM), 1,2-dichloroehane (DCE), methanol, ethanol, cyclohexanone, phosphorus tribromide, 4-(diethylamino) salicylaldehyde, cesium carbonate, 4-diethylaminoketo acid, concentrated sulfuric acid, perchloric acid, N, N-dimethylformamide, sodium bicarbonate, sodium chloride, anhydrous sodium sulfate and dimethyl sulfoxide were purchased from Energy Chemical unless otherwise stated. 3-(4,5-Dimethylthiazol-2-yl)-2,5-diphenyltetrazolium bromide (MTT), lipopolysaccharide (LPS), S-adenosyl-L-methionine (SAM), and penicillin-streptomycin solution (100X) were purchased from Beyotime Institute of Biotechnology (Shanghai, China). Fetal bovine serum (FBS) and Dulbecco’s modified Eagle’s medium (DMEM) were purchased from GIBCO Co., Ltd. All cell lines were purchased from Cell Bank of Chinese Academy of Sciences (Shanghai, China). The Rh930 was synthesized according to the previous report^1^.

**Characterization**. Nuclear magnetic resonance (NMR) spectra were measured on a Bruker Avance 400 MHz NMR spectrometer (Bruker, Switzerland). Electrospray ionization mass spectra (ESI-MS) were recorded on a mass spectrometer (Q-Exactive, USA). Ultraviolet−visible−near-infrared (UV−vis−NIR) spectra were obtained on a Lambda 1050+ spectrometer (PerkinElmer, USA). NIR-II FL spectra were obtained using an FLS1000 (Edinburgh Instruments, UK). NIR-II FL imaging was performed on Princeton Instrument NIR640 (USA).

**Calculating NIR-II quantum yield**. The quantum yield of FRHS was calculated according to our previous report^2^. The quantum yield of FRHS in DCM was calculated by using IR-26 as reference (quantum yield is 0.05% in DCE). Briefly, a series of solutions with OD < 0.1 at 1064 nm of FRHS and IR-26 were prepared, respectively. Then, the fluorescence intensities were obtained by Princeton hyperspectral imaging system (NIRvana TE640 + IsoPlane 320) under 1064 nm excitation. The integrated emission intensity (> 1100 nm) was plotted as a function of the OD value at 1064 nm. The obtained two slopes of FRHS and IR-26 were used to calculate the quantum yield of FRHS in DCM based on following equation:

**Supplementary Equation (1)**: $\mathrm{QY}_{\mathrm{sample}}= \mathrm{QY}_{\mathrm{ref}} \times\frac{n_{\mathrm{sample}}^{2}}{n_{\mathrm{ref}}^{2}} \times\frac{\mathrm{Slope}_{\mathrm{sample}}}{\mathrm{Slope}_{\mathrm{ref}}}$

Where QY_sample_ is the quantum yield of FRHS in DCM, QY_ref_ is the quantum yield of IR-26 in DCE, n_sample_ and n_ref_ are the refractive indices of DCM and DCE, respectively.

**Histological studies.** After two weeks of tumor volume monitoring and in vivo toxicity examination, all the mice were euthanized, and the major organs and subcutaneous tumors were collected and washed with PBS for three times, followed by fixing in 4% fixative solution (Cat. no. P1110, Solarbio) and embedding in paraffin. Subsequently, the paraffin-embedded tumor and organ sections were cut using a microtome (Leica RM2235, Germany) and mounted on slides. Finally, H&E-stained (G1005, Servicebio) organs and tumor sections were stained via standard histological techniques. For Ki-67 staining, the operational process was same as above. The slices were imaged by Virtual slide microscope (Olympus VS120, Japan).

**Blood biochemistry analysis**. After two weeks of PBS and FRHS treatments, the blood of the mice was collected for alanine transaminase (ALT), aspartate aminotransferase (AST), blood urea nitrogen (BUN) and creatinine (CREA) analysis.

**Statistical analysis**. All data represent the mean ± SD. One-way ANOVA with Tukey’s multiple comparisons was used for multiple comparisons when more than two groups were compared, and one-tailed or two-tailed Student’s t-test was used for two-group comparisons. All statistical differences were calculated by using GraphPad Prism 9.5 (GraphPad Software, Inc., CA, USA). In all types of statistical analysis values of P<0.05 were considered significant.

**Supplementary Figures**


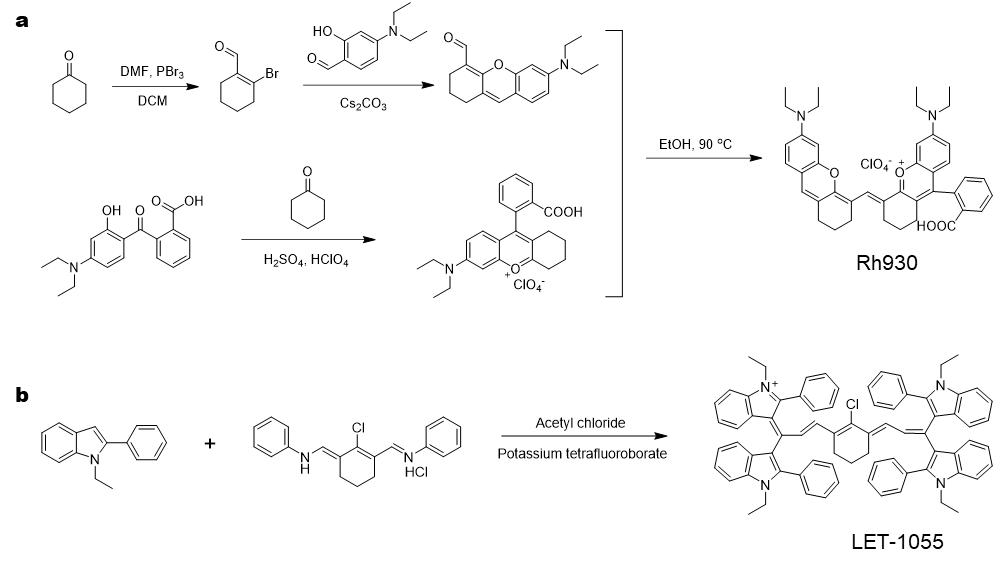


**Figure S1**. Synthetic routes of Rh930 **(a)** and LET-1055 **(b)**.


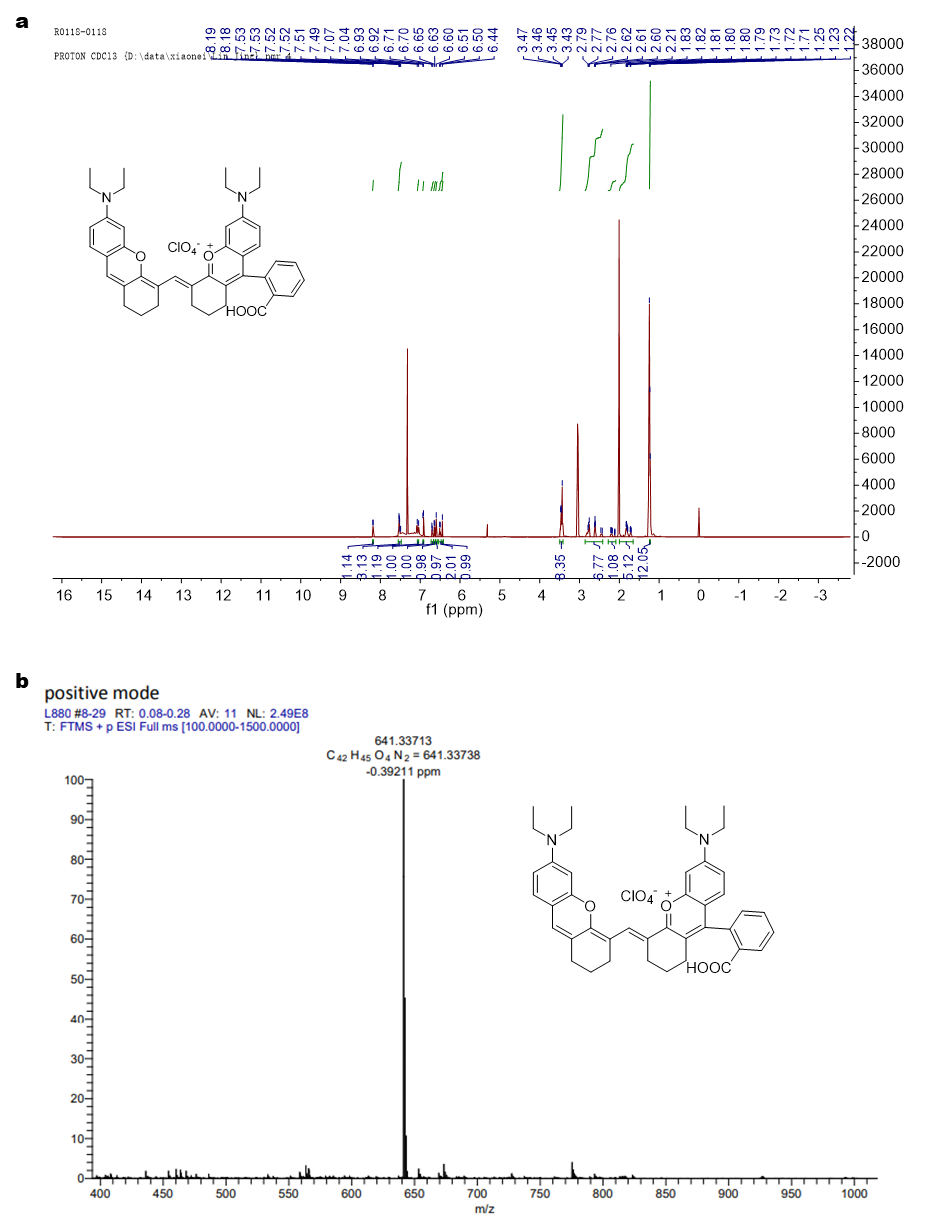


**Figure S2**. ^1^H NMR (CDCl_3_) **(a)** and ESI-MS **(b)** spectra of Rh930.


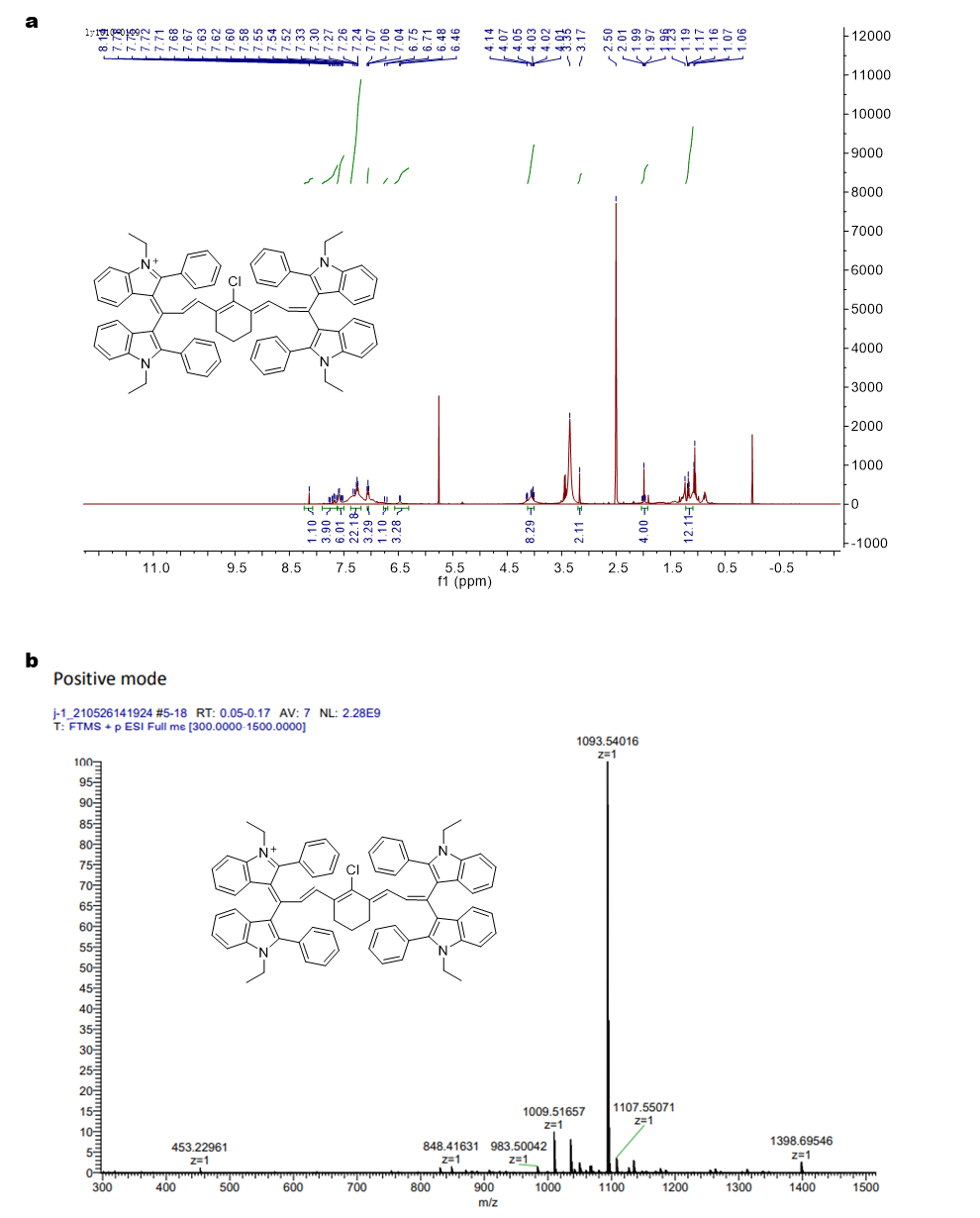


**Figure S3**. ^1^H NMR (DMSO-*d*_6_) **(a)** and ESI-MS **(b)** spectra of LET-1055.


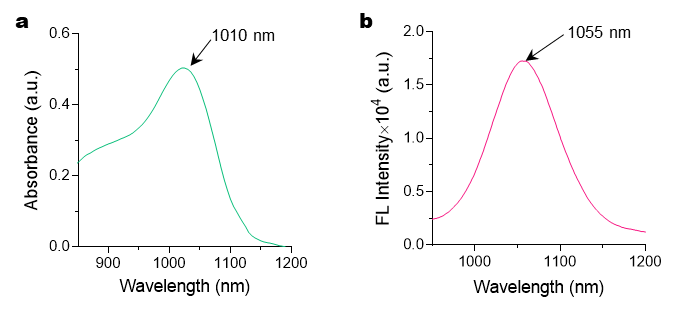


**Figure S4**. (a) UV-Vis-NIR and (b) NIR-II FL spectra of LET-1055.


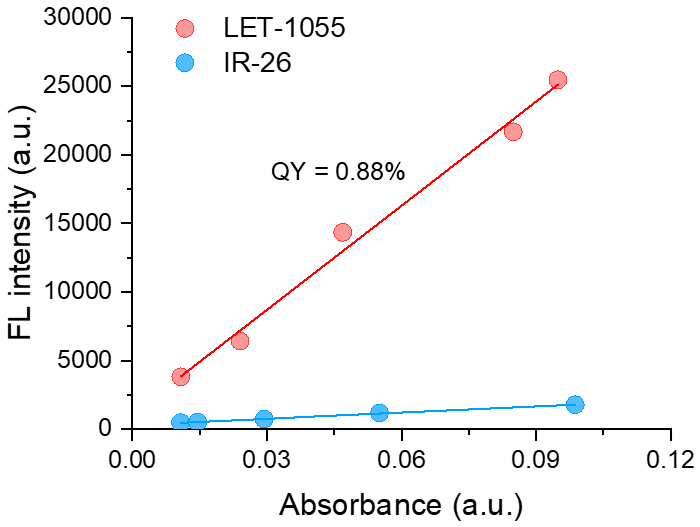


**Figure S5**. Plot of the integrated FL spectra of LET-1055 and IR-26 at five different concentrations. Linear fits were used to calculate QY by comparing the slopes to reference IR-26 (QY = 0.05%).


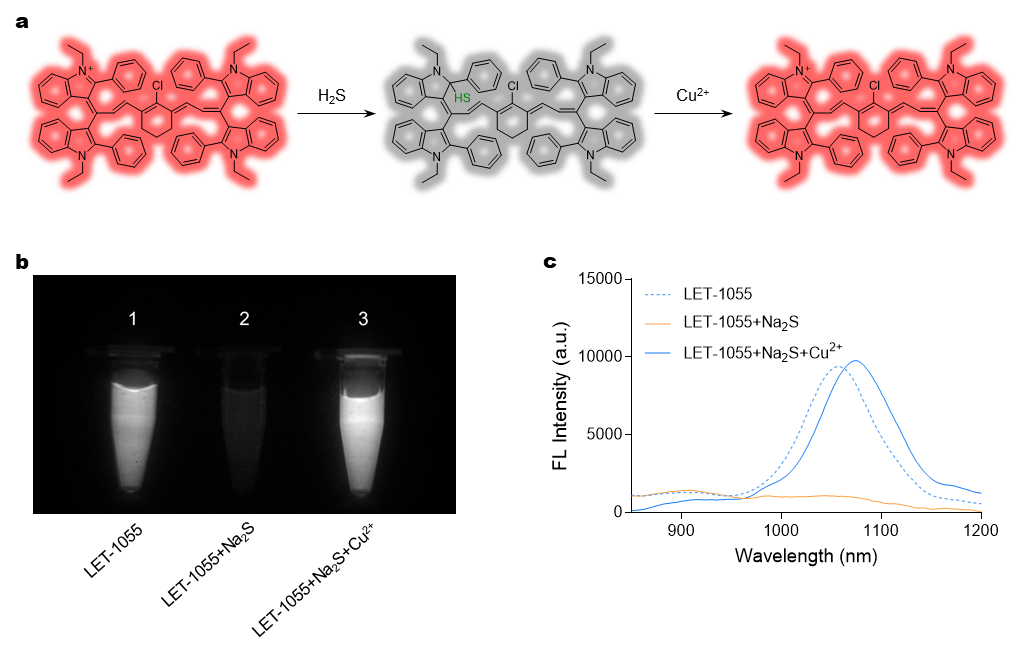


**Figure S6**. **(a)** The proposed reaction mechanism of LET-1055 with H_2_S. **(b)** NIR-II FL images of LET-1055 solution after different treatments. **(c)** NIR-II FL spectra of LET-1055 solution after different treatments. n=3 independent experiments.


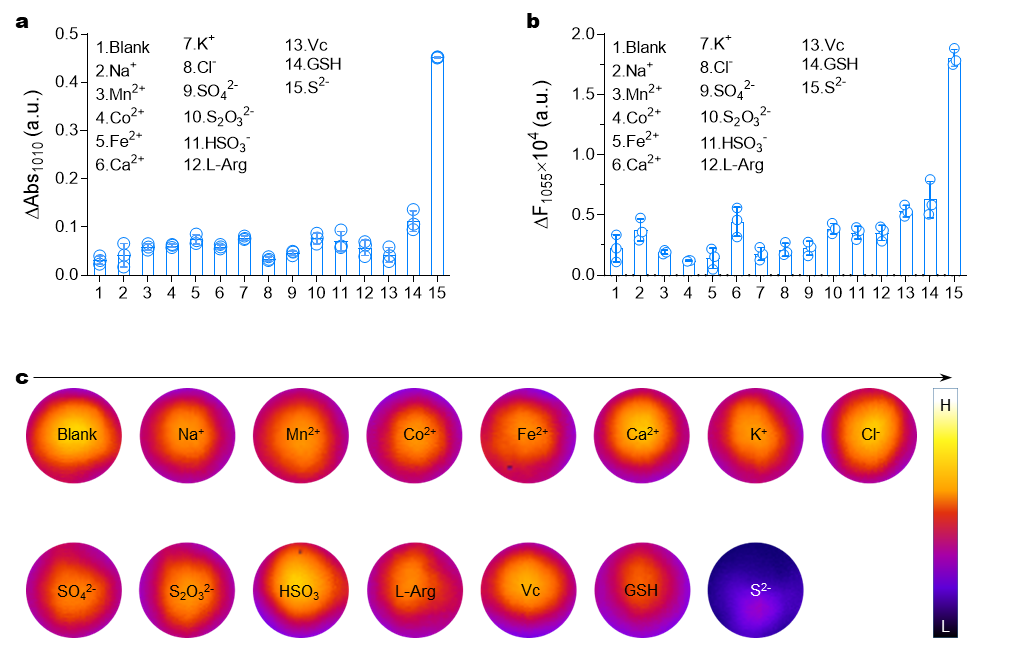


**Figure S7**. NIR-II absorbance (1010 nm) **(a)** and FL emission (1055 nm) **(b)** of LET-1055 (10 μM) upon incubation with different species (Na^+^ (500 μM), Mn^2+^ (500 μM), Co^2+^ (500 μM), Fe^2+^ (500 μM), Ca^2+^ (500 μM), K^+^ (500 μM), Cl^-^ (500 μM), SO_4_^2-^ (500 μM), S_2_O_3_^2-^ (500 μM), HSO_3_^-^ (500 μM), L-Arginine (L-Arg, 500 μM), Vitamin C (Vc, 500 μM), glutathione (GSH, 500 μM), S^2-^ (10 μM)) for 20 min. **(c)** Corresponding NIR-II FL images in **(b)**. LP: 1100 nm. Laser irradiation: 808 nm, 1 W cm^-2^. Data are presented as mean ± SD. (n = 3).


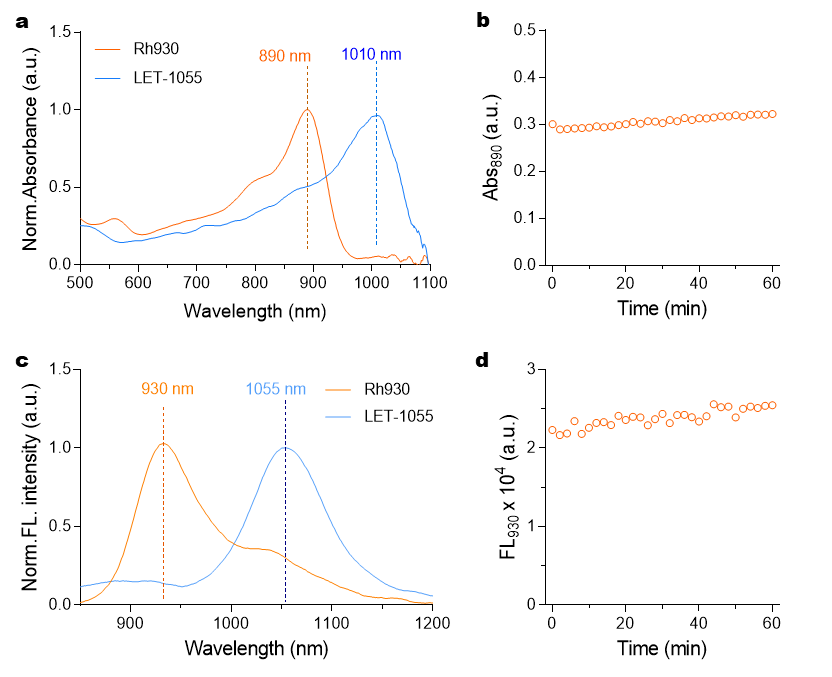


**Figure S8**. **(a)** UV-Vis-NIR absorption spectra of Rh930 and LET-1055. **(b)** Absorption intensity of Rh930 at 890 nm (Abs_890_) upon incubation with Na_2_S (10 μM) for 60 min. **(c)** NIR-II FL spectra of Rh930 and LET-1055. **(d)** FL intensity of Rh930 at 930 nm (FL_930_) upon incubation with Na_2_S (10 μM) for 60 min.


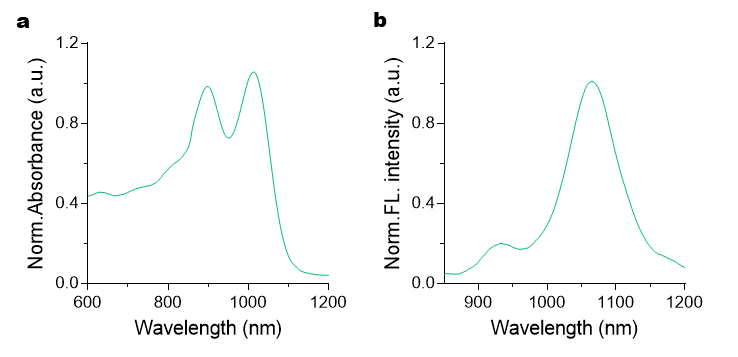


**Figure S9**. UV-Vis-NIR absorption **(a)** and NIR-II FL **(b)** spectra of FRHS.


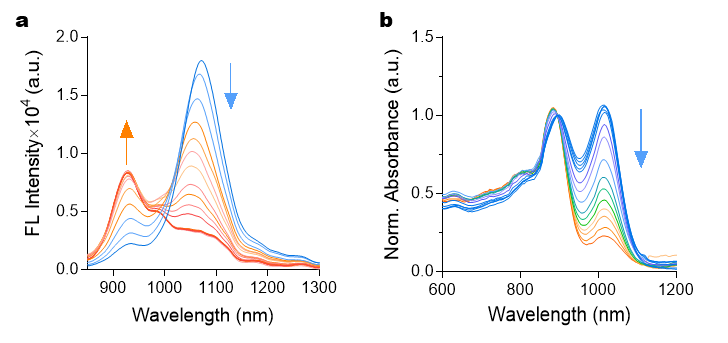


**Figure S10**. Time-dependent NIR-II FL **(a)** and normalized NIR-II absorption **(b)** spectra of FRHS after incubation with Na_2_S (10 μM) for 20 min.


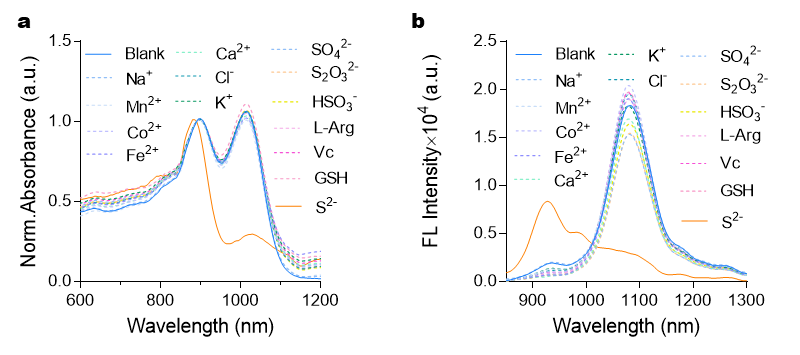


**Figure S11**. Normalized NIR-II absorption **(a)** and NIR-II FL **(b)** spectra of FRHS after incubation with different spices (Na^+^ (500 μM), Mn^2+^ (500 μM), Co^2+^ (500 μM), Fe^2+^ (500 μM), Ca^2+^ (500 μM), K^+^ (500 μM), Cl^-^ (500 μM), SO_4_^2-^ (500 μM), S_2_O_3_^2-^ (500 μM), HSO_3_^-^ (500 μM), L-Arginine (L-Arg, 500 μM), Vitamin C (Vc, 500 μM), glutathione (GSH, 500 μM), S^2-^ (20 μM)) for 20 min.


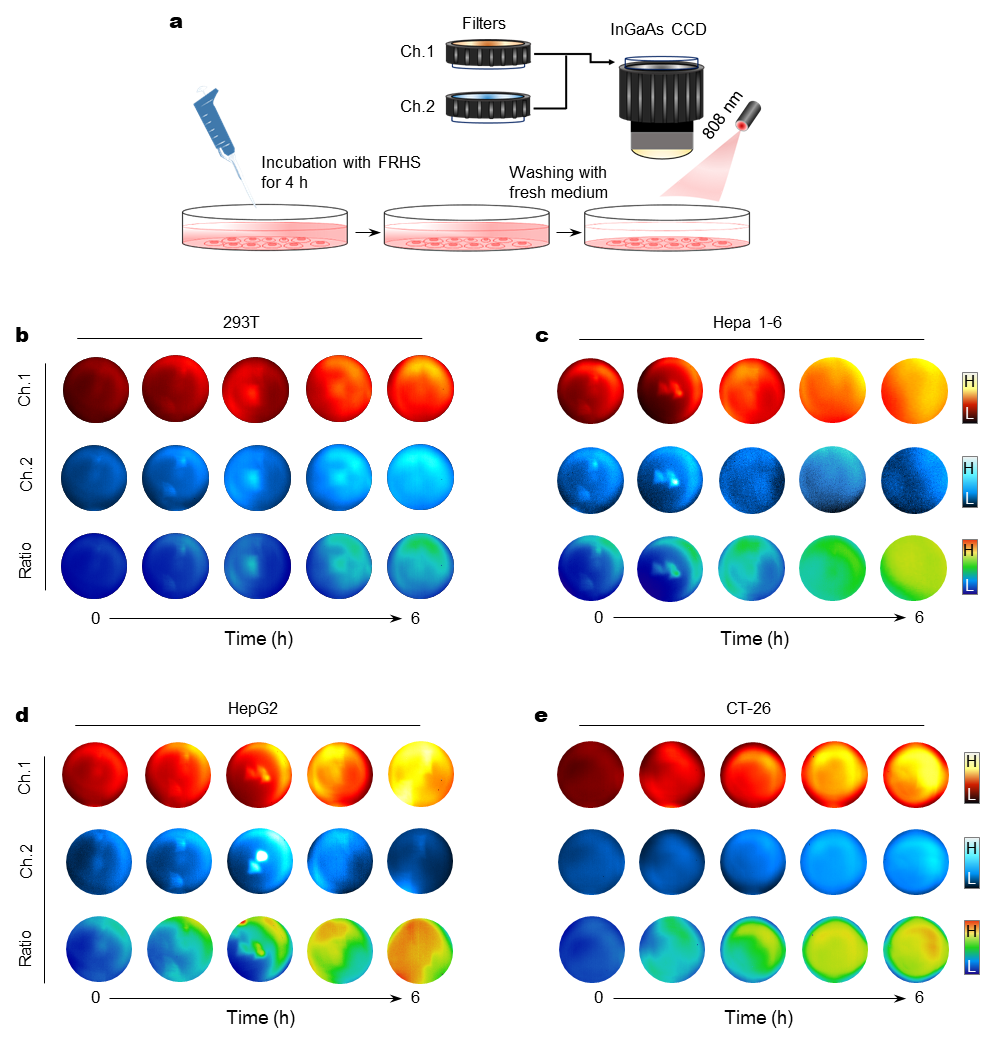


**Figure S12**. **(a)** Schematic illustration of FRHS for ratiometric NIR-II FL imaging of H_2_S in vitro. NIR-II FL images at Ch.1 and Ch.2, and ratiometric Ch.1/Ch.2 FL images of 293T **(b)**, Hepa 1-6 **(c)**, HepG2 **(d)** and CT-26 **(e)** cells after incubation with FRHS (containing 20 μM of LET-1055) for varied time. Ch.1: 900-1000 nm, filters: short-pass (SP) 1000 nm and Long-pass (LP) 900 nm. Ch.2: filters, Long-pass 1100 nm. n=3 independent experiments.


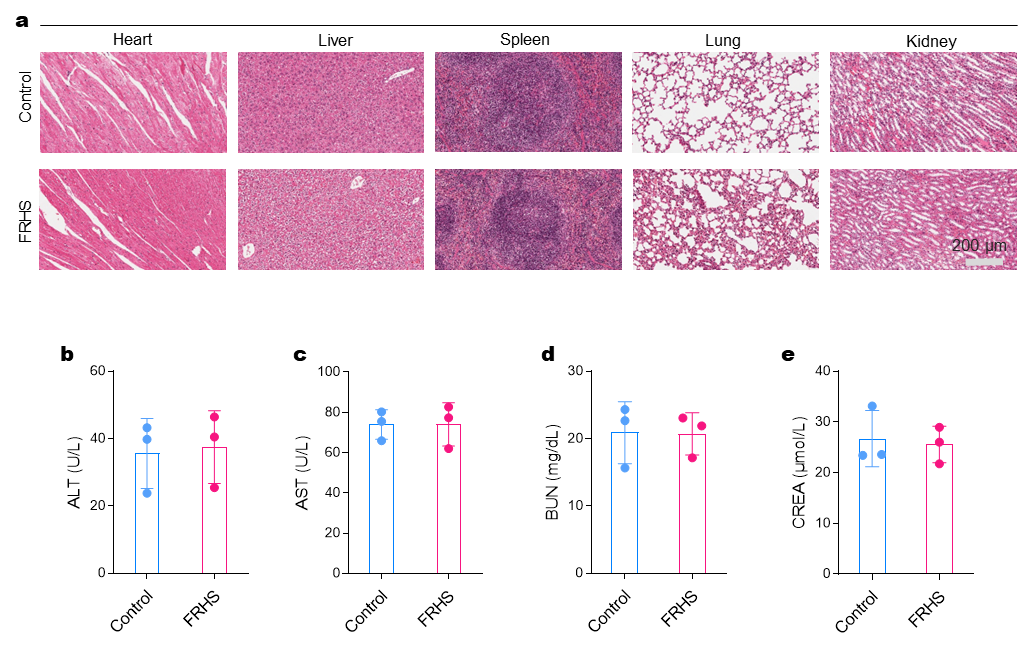


**Figure S13**. **(a)** H&E stained images of major organs (heart, liver, spleen, lung and kidney) after different treatments. n=3 independent experiments. Scale bar is 200 μm. **(b, c, d, e)** Blood biochemistry analysis (ALT, AST, BUN, and CREA) of health mice after intravenously injected with PBS or FRHS for 14 days. Data are presented as mean ± SD. (n = 3).


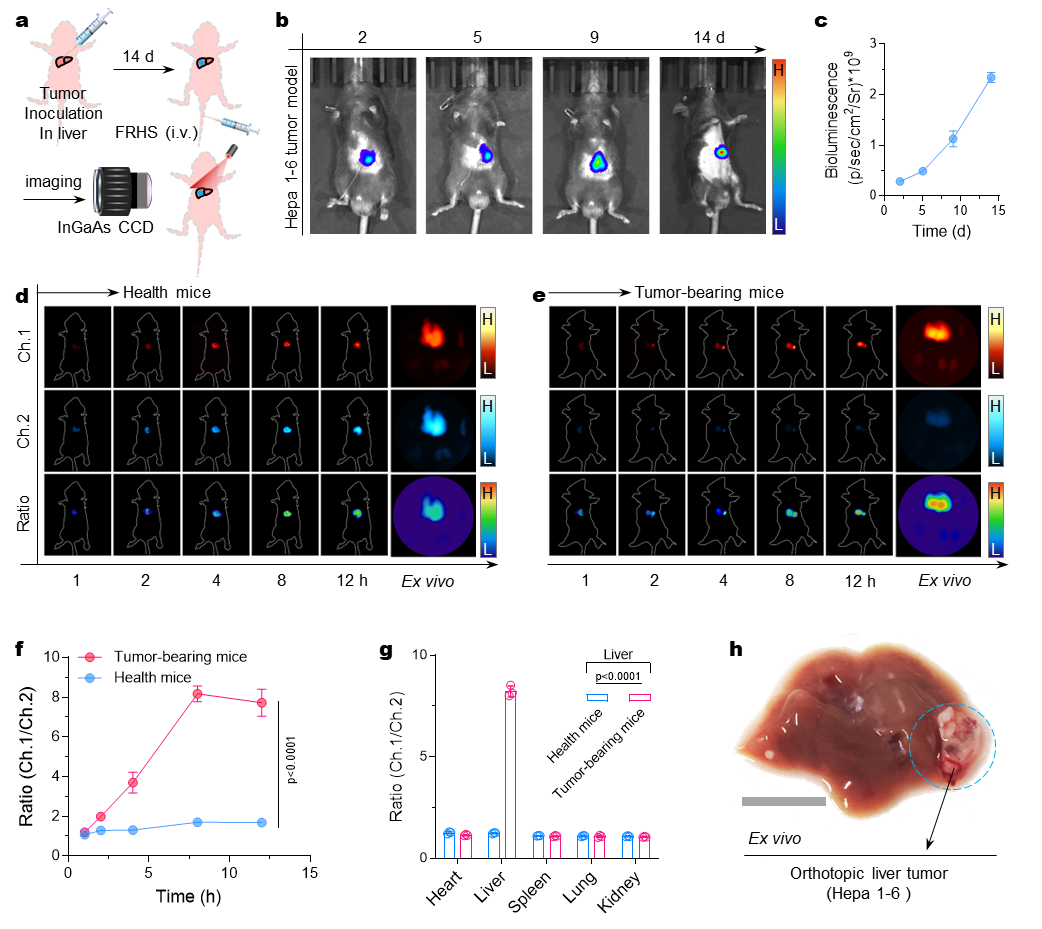


**Figure S14**. **(a)** Schematic illustration for establishment of the orthotopic liver tumor-bearing mouse model and imaging experiment. **(b)** Bioluminescence images of luciferase-transfected Hepa 1-6 cells (Hepa 1-6/Luc) inoculated mice at different time points (2, 5, 9 and 14 d). **(c)** Quantification of bioluminescence signals from (b). Data are presented as mean ± SD. (n= 3). **(d, e)** NIR-II FL images at Ch.1 and Ch.2, and ratiometric Ch.1/Ch.2 FL images of health mice **(d)** and tumor-bearing mice **(e)**, and corresponding ex vivo organs. **(f, g)** Quantification of ratiometric Ch.1/Ch.2 signals of mice after various treatments in vivo **(f)**, and ex vivo organs **(g)**. Data are presented as mean ± SD. (n= 3). Statistical significance was calculated via two-tailed Student’s t test. **(h)** Optical image of ex vivo liver (bule dashed circles: orthotopic liver tumor nodules). Scale bar is 1 cm. n=3 independent experiments.


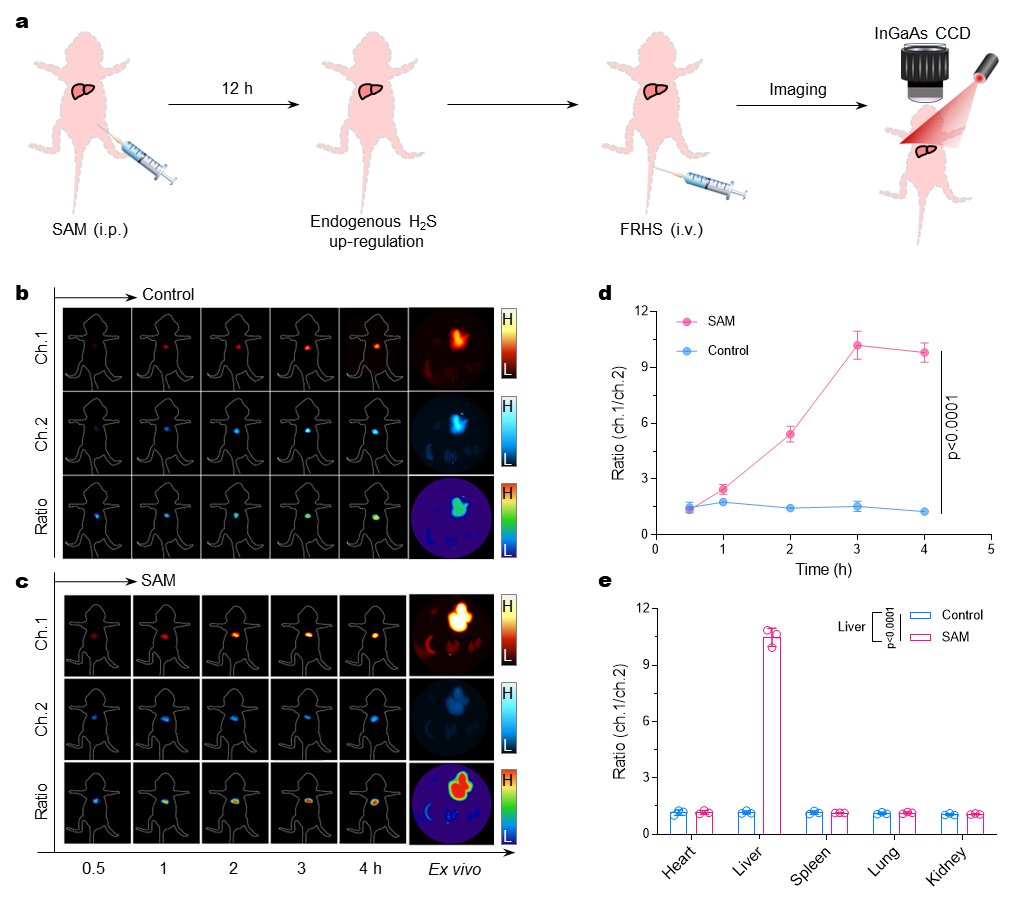


**Figure S15**. **(a)** Schematic illustration for establishment of the liver-injured mouse model and imaging experiment. **(b, c)** NIR-II FL images at Ch.1 and Ch.2, and ratiometric Ch.1/Ch.2 FL images of mice with PBS **(b)** and SAM **(c)** treatments, and the corresponding ex vivo organs. **(d, e)** Quantification of ratiometric Ch.1/Ch.2 signals of mice after various treatments in vivo **(d)**, and ex vivo organs **(e)**. Data are presented as mean ± SD. (n= 3). Statistical significance was calculated via two-tailed Student’s t test.

**References**

(1) Lei, Z.; Sun, C.; Pei, P.; Wang, S.; Li, D.; Zhang, X.; Zhang, F., Stable, Wavelength-Tunable Fluorescent Dyes in the NIR-II Region for In Vivo High-Contrast Bioimaging and Multiplexed Biosensing. *Angew. Chem. Int. Ed.* **2019,** *58* (24), 8166-8171.

(2) Li, B.; Liu, H.; He, Y.; Zhao, M.; Ge, C.; Younis, M. R.; Huang, P.; Chen, X.; Lin, J., A "Self-Checking" pH/Viscosity-Activatable NIR-II Molecule for Real-Time Evaluation of Photothermal Therapy Efficacy. *Angew. Chem. Int. Ed.* **2022,** *61* (16), e202200025.
